# Patterns of neighborhood environment attributes related to physical activity across 11 countries: a latent class analysis

**DOI:** 10.1186/1479-5868-10-34

**Published:** 2013-03-14

**Authors:** Marc A Adams, Ding Ding, James F Sallis, Heather R Bowles, Barbara E Ainsworth, Patrick Bergman, Fiona C Bull, Harriette Carr, Cora L Craig, Ilse De Bourdeaudhuij, Luis Fernando Gomez, Maria Hagströmer, Lena Klasson-Heggebø, Shigeru Inoue, Johan Lefevre, Duncan J Macfarlane, Sandra Matsudo, Victor Matsudo, Grant McLean, Norio Murase, Michael Sjöström, Heidi Tomten, Vida Volbekiene, Adrian Bauman

**Affiliations:** 1 Exercise and Wellness, School of Nutrition and Health Promotion, Arizona State University, Phoenix, AZ, USA; 2 Active Living Research, University of California, San Diego, CA, USA; 3 Risk Factor Monitoring and Methods Branch, Applied Research Program, National Cancer Institute, National Institutes of Health, Bethesda, MD, USA; 4 Unit for Preventive Nutrition, Department of Biosciences and Nutrition, Karolinska Institutet, Stockholm, Sweden; 5 School of Education, Psychology and Sports Science, Linnaeus University, Kalmar, Sweden; 6 School of Population Health, The University of Western Australia, Crawley, WA, Australia; 7 Canadian Fitness and Lifestyle Research Institute, School of Public Health, Ottawa, Canada; 8 Valnesfjord Rehabilitation Centre, Valnesfjord, Norway; 9 Sport New Zealand, Ministry of Health, Wellington, New Zealand; 10 Department of Movement and Sport Sciences, Ghent University, Ghent, Belgium; 11 Pontificia Universidad Javeriana, Bogota, Colombia; 12 Department of Preventive Medicine and Public Health, Tokyo Medical University, Tokyo, Japan; 13 Department of Kinesiology, Katholic University, Leuven, Belgium; 14 Institute of Human Performance, The University of Hong Kong, Hong Kong, China; 15 Center of Studies of the Physical Fitness Research Center from São Caetano do Sul, CELAFISCS, Sao Paulo, Brazil; 16 Oppegård Municipality, Oppegård County, Norway; 17 Department of Sport Science, Lithuanian Academy of Physical Education, Kaunas, Lithuania; 18 Prevention Research Collaboration, University of Sydney, Sydney, Australia

**Keywords:** Built environment, International, Recreation, Surveillance, Exercise

## Abstract

**Background:**

Neighborhood environment studies of physical activity (PA) have been mainly single-country focused. The International Prevalence Study (IPS) presented a rare opportunity to examine neighborhood features across countries. The purpose of this analysis was to: 1) detect international neighborhood typologies based on participants’ response patterns to an environment survey and 2) to estimate associations between neighborhood environment patterns and PA.

**Methods:**

A Latent Class Analysis (LCA) was conducted on pooled IPS adults (N=11,541) aged 18 to 64 years old (mean=37.5 ±12.8 yrs; 55.6% women) from 11 countries including Belgium, Brazil, Canada, Colombia, Hong Kong, Japan, Lithuania, New Zealand, Norway, Sweden, and the U.S. This subset used the Physical Activity Neighborhood Environment Survey (PANES) that briefly assessed 7 attributes within 10–15 minutes walk of participants’ residences, including residential density, access to shops/services, recreational facilities, public transit facilities, presence of sidewalks and bike paths, and personal safety. LCA derived meaningful subgroups from participants’ response patterns to PANES items, and participants were assigned to neighborhood types. The validated short-form International Physical Activity Questionnaire (IPAQ) measured likelihood of meeting the 150 minutes/week PA guideline. To validate derived classes, meeting the guideline either by walking or total PA was regressed on neighborhood types using a weighted generalized linear regression model, adjusting for gender, age and country.

**Results:**

A 5-subgroup solution fitted the dataset and was interpretable. Neighborhood types were labeled, “Overall Activity Supportive (52% of sample)”, “High Walkable and Unsafe with Few Recreation Facilities (16%)”, “Safe with Active Transport Facilities (12%)”, “Transit and Shops Dense with Few Amenities (15%)”, and “Safe but Activity Unsupportive (5%)”. Country representation differed by type (e.g., U.S. disproportionally represented “Safe but Activity Unsupportive”). Compared to the Safe but Activity Unsupportive, two types showed greater odds of meeting PA guideline for walking outcome (High Walkable and Unsafe with Few Recreation Facilities, OR= 2.26 (95% CI 1.18-4.31); Overall Activity Supportive, OR= 1.90 (95% CI 1.13-3.21). Significant but smaller odds ratios were also found for total PA.

**Conclusions:**

Meaningful neighborhood patterns generalized across countries and explained practical differences in PA. These observational results support WHO/UN recommendations for programs and policies targeted to improve features of the neighborhood environment for PA.

## Introduction

Non-communicable diseases (NCDs) including cardiovascular diseases, type 2 diabetes, and cancers represent a growing global health burden, of which the greatest increases are expected in low and middle-income countries [1]. Physical inactivity, the 4^th^ leading cause of death worldwide, is an important modifiable risk factor of several NCDs [2,3], and is occurring at epidemic rates in urbanized regions worldwide [4]. The World Health Organization (WHO) and the United Nations (UN) have recommended strategies for preventing NCDs including policies to improve urban design, public transportation, and recreation facilities that can encourage large portions of the population to be physically active [1,5].

Over the last decade, the literature relating attributes of neighborhood environments and physical activity has grown, with most studies conducted in regions within a single country, particularly the U.S., Australia, and a few European nations [6]. These studies found that “walkable neighborhoods” characterized by high residential density, well connected grid-like street networks, and accessible and diverse destinations within walking distance [7-9] were associated with active transportation, particularly walking for transport [10-12]. Additionally, access to parks and recreation facilities shows significant associations with recreational physical activity [13-15]. The presence of existing and new public transportation options in neighborhoods appears associated with greater physical activity [16,17]. Studies on neighborhood crime, traffic safety and physical activity had inconsistent findings [18].

Although there has been overall support for the associations of neighborhood environments and physical activity, the current evidence is not as strong and consistent as expected [19-21]. Several methodological limitations might have contributed to the large number of null associations in the literature. For example, a conceptual mismatch between the attributes of the built environment and the domain of physical activity may lead to small, unexpected or non-significant associations. Additionally, since most studies were conducted in regions within single countries, limited variability in neighborhood environments might lead to underestimation of the environment--physical activity association [6,22]. Moreover, neighborhood environments include a multitude of attributes that can correlate in complex ways (i.e. not independent). It is a challenge to examine concurrent associations of neighborhood attributes with physical activity since simply adjusting for other attributes to isolate a single variable may lead to over-adjustment and type 2 errors.

A more suitable approach for evaluating environmental variables that tend to co-occur is to identify neighborhood typologies. Several approaches could be used to examine numerous variables and complex neighborhood environment relationships. One approach is to develop an index from disparate but conceptually linked items, such as a walkability index [8]. Another approach is to factor analyze to examine how items from measures of the built environment may combine empirically to form one or more underlying independent uni-dimensional factors [23]. A third approach is to examine interactions between several items (e.g. access to parks by intersection density), but this approach can become difficult to interpret for second order and higher interactions and requires large samples. Finally, latent class analysis is a multivariate approach useful for identifying common patterns among numerous variables and classifying individuals into subgroups based on their response patterns. This latter approach could be used to examine combinations of disparate neighborhood attributes conceptually related to physical activity [14], and only recently has been used to examine built environment relations [24-28].

The International Prevalence Study (IPS) presented an opportunity for such inquiry while improving upon prior studies by examining multiple environmental attributes from large samples in multiple countries. The IPS was a collaborative cross-sectional study that aimed to collect representative data from 20 diverse countries to compare physical activity prevalence rates internationally. Previous IPS publications have reported on the prevalence of physical activity across countries and the bivariate relations between environmental items and physical activity [29,30]. The purpose of the current analysis among the pooled IPS sample was to: 1) examine whether unique neighborhood typologies, based on IPS participants’ response patterns, could be derived from a brief surveillance survey of environment attributes, 2) to classify participants into neighborhood type subgroups based upon detected response patterns, and 3) estimate associations between detected neighborhood types and physical activity. It was hypothesized that participant response patterns would result in a differentiation of neighborhood types prevalent across a diverse range of countries, and that derived patterns indicating combined presence and/or the highest probability of all the supportive neighborhood attributes would be related to the highest levels of total physical activity, while those indicating the combined absence and/or a low probability of each attribute would be unsupportive for total physical activity.

## Methods

The IPS study design and sampling approach has been described previously [29]. Briefly, international investigators were invited to participate based on their interest and capacity to adhere to study guidelines and protocols. The study sample in each country was intended to be representative of the national population, or of a significant region or city (defined as greater than 1 million people) within a country. The IPS sampling and recruitment protocols were established and measures standardized with some allowances to accommodate local circumstances in countries. In each country, households were mainly selected at random and individuals within households selected either by most recent birthday or randomly. Data collection occurred during spring and fall seasons of 2002 and 2003; these seasons were judged to be relatively comparable across countries. Surveys were conducted by telephone, face-to-face, or by self-administration. The current analyses considered adults aged 18 to 64 years old from 11 of the 20 countries including Belgium, Brazil, Canada, Colombia, Hong Kong (SAR, China), Japan, Lithuania, New Zealand, Norway, Sweden, and the United States because these countries included measures of both physical activity and the built environment (an optional measure) as part of their survey. Each country provided a statement of ethics approval from their research center/institution, and all study participants provided informed consent either verbally or in written form.

### Physical Activity Neighborhood Environment Survey (PANES)

The PANES is a brief survey developed specifically for the IPS and previously named the IPS Environmental Module. The PANES is a 17-item questionnaire (7 core and 10 optional items) that measures attributes of the neighborhood built and social environments hypothesized, or known, to be related to physical activity [15]. Neighborhood was defined as the “area all around your home that you could walk to in 10–15 minutes.” Only the 7 core items were asked by all 11 countries. Core items used a single question to assess each of the following attributes: residential density; access to shops/services, public transit, and recreation facilities; presence of sidewalks, bike paths; and personal safety from crime.

Core items, except for residential housing type, were asked using a 4-point Likert-type scale ranging from “strongly disagree” to “strongly agree”. The housing type question was asked using a 5-point scale that ranged from “Detached single-family housing” indicating low residential density to “Apartments or condos of more than 12 stories” indicating high residential density. Core items presented both questions and statements including, 1) “What is the main type of housing in your neighborhood?”, 2) “Many shops, stores, markets or other places to buy things I need are within easy walking distance of my home.”, 3) “It is within a 10–15 minutes walk to a transit stop (such as bus, train, trolley, or tram) from my home.”, 4) “There are sidewalks on most of the streets in my neighborhood.”, 5) “There are facilities to bicycle in or near my neighborhood, such as special lanes, separate paths or trails, shared use paths for cycles and pedestrians.”, 6) “My neighborhood has several free or low cost recreation centers, playgrounds, public swimming pools, etc.”, and 7) “The crime rate in my neighborhood makes it unsafe to go on walks at night.” Except for the housing type variable, response options were collapsed and recoded for data analysis into a 2-level variable: disagree (strongly disagree and somewhat disagree) vs. agree (strongly agree and somewhat agree). Housing type was recorded to a 2-level variable to compare “Single detached-family housing” (i.e., low density) to the 4 other housing types (i.e., high density), with high density conceptually related to more physical activity based on previous positive correlations with total activity and total walking measures [10,31]. Personal safety was reverse-scored so that higher values indicated a safer neighborhood. Dichotomized PANES items have shown fair-to-substantial reliability (Kappas = 0.35 to 0.70) and acceptable validity (rho = 0.31 to 0.81) compared to multi-item subscales from another validated and often-used built environmental measure [32]. Reliability of PANES also has been documented in Sweden and Nigeria [33,34]. The PANES is available at: http://sallis.ucsd.edu.

### International Physical Activity Questionnaire (IPAQ)

In each country, participants’ walking, moderate, and vigorous intensity physical activities over the last 7 days were measured using the IPAQ short form. Participants were asked to report the frequency and usual duration of each type of activity performed for at least 10 minutes at a time. The IPAQ has undergone cultural adaptation and translation for use across countries. The reliability and validity of the IPAQ were evaluated internationally, and it performed similarly to other self-reported physical activity measures when compared to accelerometers (ICC = 0.7-0.8, rho = 0.3) [35]. IPAQ survey and cultural adaptation and scoring protocols are available online at: http://www.ipaq.ki.se.

The IPAQ was scored to determine whether participants engaged in adequate amounts of moderate-to-vigorous physical activity (MVPA) to meet physical activity recommendations. Meeting the physical activity recommendation [36] was operationalized in two ways: 150 minutes of physical activity per week accumulated through walking only, and through any combination of walking, moderate and vigorous activities. Estimates of meeting the guidelines were calculated as the sum of the reported frequency multiplied by the typical duration of each intensity category.

### Demographics

Participants reported demographic characteristics with age and gender being shared variables in all 11 countries.

## Statistical analyses

Descriptive statistics for demographic, environmental and physical activity variables were reviewed by country. Participants living in areas with a population <30,000 were excluded from further analyses because the PANES was not developed for or validated in rural areas. Afterwards, datasets from each country were pooled for further analyses.

A Latent Class Analysis (LCA) was conducted using the seven PANES core items in Mplus version 6.0. The goal of a LCA was to derive meaningful classes from a sample, assign participants to classes, and explore class associations with physical activity. Conceptually, LCA is a special case of finite mixture modeling operationalized by dichotomous indicator variables and a categorical latent variable. LCA derives mutually exclusive classes that maximize between-group variance and minimize within-group variance based on several model fit criteria [37]. Subgroups within a sample are detected by the most frequent response patterns derived by participants’ responses to the 7 environmental variables, against background noise that can result from measurement error. For 7 items on a dichotomous scale, 128 (i.e., 2^7^) response patterns are possible with different sample sizes per pattern.

To identify the ideal number of classes in the pooled sample, solutions of 1 to 8 classes were tested. Model fit criteria were evaluated across solutions to determine the number of latent classes that best represented the data. These criteria included model convergence, Akaike information criterion (AIC) [38], Bayesian information criterion (BIC) [39], sample sizes per class, and interpretability. Item-response probabilities of classes were charted for visual interpretation. Item-response probabilities show the probability of an affirmative response to each variable conditional on the latent classes. Robust standard errors for item-response probabilities were calculated using sandwich estimators. Individuals were given a probability of being part of each derived class and classified post-analysis into one of the derived classes using their highest posterior probability. The mean maximum posterior probabilities per latent class were calculated by averaging the maximum posterior probabilities for individuals classified to a specific class. Mean values close to 1 indicate a strong degree of homogeneity and classification certainty, the most important characteristics of a good latent class model [37]. The class prevalences and item response probabilities were presented by latent class. Country variation in latent classes was charted to show country representation.

To estimate associations between derived latent neighborhood patterns (classes) and physical activity, a weighted (normalized) generalized linear regression model specified with a binomial distribution and logit link function was used to regress binary variables, meeting physical activity guidelines by walking only or through a combination of activities, on the derived latent class variables independently after adjusting for the covariates of sex, age and country (as a nested variable) in SPSS version 18. Educational attainment was available for 9 countries only and was not included as a covariate. Log odds and 95% confidence intervals of meeting guideline were antilogged and presented by latent class in the text and figures. All analyses were performed between 2011 and 2012.

## Results

Complete survey data were available for 11,541 adults; Belgium (n=357), Brazil (n=876), Canada (n=619), Colombia (n=2674), Hong Kong (n=990), Japan (n=442), Lithuania (n=1291), New Zealand (n=803), Norway (n=492), Sweden (n=434), and the United States (n=2563), and were included in the latent class analysis. Demographic characteristics for participants by country have been presented previously [29,30]. For the pooled analytic sample, participants with complete survey data ranged from 18 to 64 years of age (M=37.5 SD±12.8) and 55.6% were women.

Table 1 presents model fit criteria for latent class solutions. An 8-class solution did not result in statistical convergence, and the 7- and 6-class solutions resulted in diminishing gains on model fit criteria (e.g. AIC, BIC, and adjusted BIC) and convoluted interpretations. A 5-class solution was deemed to best fit the dataset based on adjusted BIC, BIC, and AIC model fit criteria and interpretability of classes. The mean maximum posterior probabilities for the 5 classes were 0.82, 0.74, 0.75, 0.81, and 0.79 for classes 1 to 5 respectively, providing evidence of homogeneity for each subgroup.

**Table 1:** Model fit indices for latent class analysis of 1 to 8 solutions

**Number of classes**	**# of estimated parameters**	**df**	**AIC**^**1**^	**BIC**^**2**^	**Adjusted BIC**
1	7	120	95331.26	95382.74	95360.49
2	15	112	91497.38	91607.69	91560.02
3	23	104	89729.21	89898.35	89825.26
4	31	96	89123.12	89351.08	89252.56
5	39	88	88775.30	89062.09	88938.15
6	47	80	88685.71	89031.33	88881.97
7	55	72	88622.30	89026.75	88851.96
8		Not specified			

^1^ Akaike Information Criterion; ^2^ Bayesian Information Criterion.

**Figure 1:**
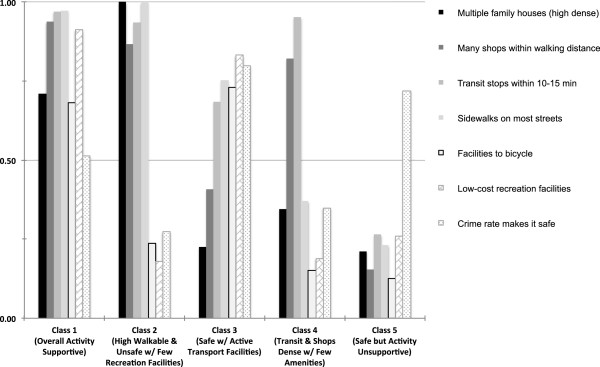
**Item-response probabilities for each environmental attribute by latent class. **^1^Higher values indicate a high probability of an affirmative response to the item. Values of .5 indicate an equal probability of affirmative and negative responses to an item. Values approaching zero indicate a low probability of an affirmative response.

Figure 1 shows item-response probabilities by latent class. Class 1 was labeled “overall activity supportive” and represented 51.6% of the pooled sample. The class was characterized by a combination of probability values that indicated a high prevalence of multiple family households (high density) and close proximity to many shops and services, transit stops, sidewalks, biking facilities, low cost recreation facilities, and a moderate probability of safety from crime. This class had the highest values relative to the other classes on shops and services and transit access, suggesting a high number of destinations, and low-cost recreation facilities. Class 2 was labeled “high walkable and unsafe with few recreation facilities” and represented 16.4% of the sample. Class 2 was characterized as such because of a combination of high probabilities of multiple family households, sidewalks, access to shops and services, and transit stops. In this class, bicycle and low-cost recreational facilities were unlikely in neighborhoods, and reported personal safety was the worst among all the classes. Class 3 represented 11.5% of the sample and was labeled “safe with active transport facilities”. This class had the highest probability of being safe from crime and having bicycle facilities relative to other classes. Class 4 represented 15.2% of the pooled sample and was labeled as “transit and shops dense with few amenities.” Class 4 had a high likelihood of transit stops, shops and services, but was generally unsafe and unlikely to have multiple family houses, sidewalks on most streets, bicycle facilities, and low cost recreation facilities within this neighborhood type. Class 5 was labeled “safe but activity unsupportive” because of the generally safe conditions but low likelihoods of any activity promoting features in this neighborhood type. Class 5 represented 5.3% of the pooled sample.

**Figure 2:**
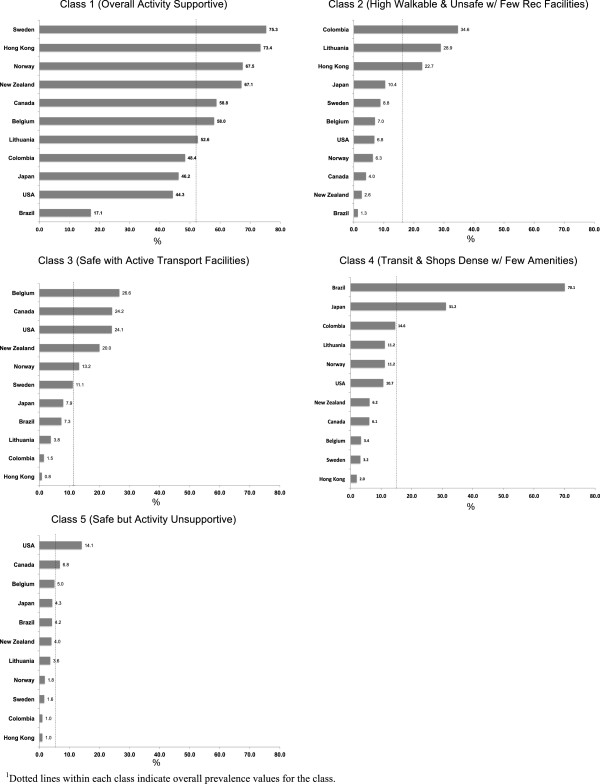
**Country-specific prevalence within each latent class. **^1^Dotted lines within each class indicate overall prevalence values for the class.

Figure 2 shows variation in classification into a specific class by country. For each class, the vertical line indicates the overall prevalence noted by the above percentages while country-specific bars show the prevalence for each country within a class. In Class 1 (overall activity supportive), prevalence was higher for Sweden, Hong Kong, Norway, New Zealand, Canada, and Belgium and lower for Brazil, United States, Japan and Colombia. In Class 2 (high walkable and unsafe with few recreation facilities) the prevalence for Colombia, Lithuania, and Hong Kong was greatest, while lower proportions occurred for the other countries, especially from Brazil and New Zealand and Canada. For Class 3 (safe with active transport facilities), very low prevalence was observed for Hong Kong and Colombia, but this class was overrepresented by those from Belgium, Canada, United States and New Zealand. Class 4 (transit and shops dense with few amenities) was overwhelmingly represented by greater proportions of participants from Brazil and Japan, while representing very low proportions from Hong Kong, Sweden, and Belgium. Class 5 (safe but activity unsupportive) was underrepresented proportionally by participants from almost all countries, especially Hong Kong, Colombia, Sweden and Norway, but was overrepresented by participants from Canada and the United States.

**Figure 3:**
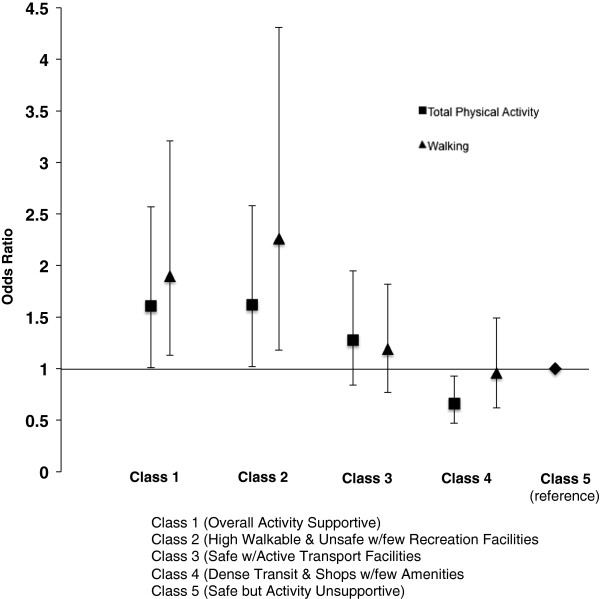
**Relative odds of meeting the 150-minute guideline for walking only and for combined walking, moderate, and vigorous activities by neighborhood latent class. **^1^Adjusting for age, sex, and participants nested within country.

Figure 3 presents a generalized linear regression model that examined the odds of meeting the physical activity guideline for walking only and total physical activity for each of the classes relative to Class 5 (safe but activity unsupportive), after adjusting for covariates. Class 5 was selected as the reference class because every neighborhood attribute (except for safety) was expected to be associated with lower physical activity levels. Compared to Class 5, two classes showed significantly greater odds and one class showed significantly lower odds of meeting the guideline for total physical activity. Participants in Class 1 (overall activity supportive) had 1.61 times the odds (95% CI 1.05-2.57) and participants in Class 2 (high walkable and unsafe with few recreation facilities) had 1.62 times the odds (95% CI 1.02-2.58) of meeting guideline for total physical activity. Participants classified in Class 4 (transit and shops dense with few amenities) were less likely (OR = 0.66, 95% CI 0.47 – 0.93) to meet the guideline for total physical activity. For meeting the guideline by walking, and compared to Class 5, two classes showed significantly greater odds of meeting the guideline. Participants in Class 2 (high walkable and unsafe with few recreation facilities) had 2.26 times the odds (95% CI 1.18-4.31) and participants in Class 1 (Overall Activity Supportive) had 1.90 times the odds (95% CI 1.13-3.21) of meeting the guideline by walking. The odds for participants in Class 4 (Dense Transit and Shops with Few Amenities) were not statistically different (OR = 0.96, 95% CI 0.62-1.49) from those in Class 5.

## Conclusions

This study of reported built environment attributes from representative samples of participants in 11 countries found that a combination of seven neighborhood environment attributes resulted in five meaningful neighborhood patterns across countries. These patterns of co-existing neighborhood attributes varied in their conceptual supportiveness of physical activity with one revealing an overall pattern of unsupportive built environment attributes, others revealing differing patterns balanced towards between either more walkable or more recreational attributes, and one pattern revealing an “overall activity supportive” pattern. Interestingly, the proportions of participants grouped in each pattern varied by country, suggesting that representation of these neighborhood types differed across countries. Only two patterns (i.e., “overall activity supportive” and “high walkable and unsafe with few recreation facilities”) were significantly associated with meeting the physical activity guideline either by walking only or total physical activities. Previous analyses of the current dataset found bivariate associations between specific supportive neighborhood attributes and physical activity levels, and a summary score suggested that the greater number of neighborhood attributes supporting physical activity the higher odds of meeting the physical activity guideline [30]. The current study adds to previous findings by answering the question “which patterns, if any, are associated with sufficient walking or total physical activity, and which patterns of amenities occur most frequently across countries?”

Combinations of neighborhood attributes revealed specific neighborhood types. Based on the hypothesis derived from ecological models, [14] it was expected that meaningful combinations of neighborhood attributes would emerge and combinations with more attributes supportive of physical activity would be associated with physical activity. A pattern defined by low probabilities of residential density, shops, sidewalks, transit stops, bicycling and recreation facilities but generally safe from crime at night was identified. This class was used as a reference category because conceptually it represented the most unsupportive pattern for physical activity (i.e., safe but activity unsupportive (Class 5)). This class had the lowest prevalence (5.3%) of participants overall, but participants from the U.S. (14.1%) were 2 times more likely to be classified into this pattern. Canada was also overrepresented, but not to the same extent as the U.S. Both countries were disproportionally overrepresented compared to other countries such as Hong Kong, Colombia, Sweden, Norway (less than 2%). The result is consistent with the history of the last 70 years in North America where many neighborhoods have been designed from a perspective that favored automobile-dependent and suburban type developments with low residential densities and segregated land uses, [40] patterns generally associated with lower PA, higher obesity rates, and other deleterious health and environmental exposure outcomes [41,42].

The pattern observed in Class 1 (overall activity supportive) showed the most favorable neighborhood design attributes with positive probabilities for all built environment attributes (crime safety was about equal). Based on a hypothesis from an ecological perspective, Class 1 was expected to have the strongest association with physical activity of all the classes. Relative to Class 5, Class 1 neighborhood pattern was strongly associated with meeting guideline for both total physical activity (OR=1.61) and walking (OR=1.90). The association for meeting the guideline in Class 1 for walking appeared stronger than for total activity, but the overlap in confidence intervals suggests that these may not be statistically different. Interestingly, the majority of participants across 11 countries were classified into this overall supportive pattern (52% prevalence). A higher prevalence of participants from many European countries, with cities designed for walking, were included in this class such as Sweden, Norway, and Belgium. Also, proportionally many participants from Hong Kong, New Zealand, and Canada were overrepresented by this class compared to the overall prevalence (52%). Participants from Brazil, the U.S., Japan, and Colombia were underrepresented compared to the overall prevalence. It was surprising that some countries were so well represented by Class 1, such as the U.S. with a prevalence of 44% when objective data for walkability suggest that such neighborhoods are uncommon in many parts of North America [43]. A representative sampling approach was undertaken in each country, so this result is difficult to explain but may reflect the difference between objective and self-reported environmental features of neighborhoods [44,45]. In general, the strong association between Class 1 and PA supports the hypothesis that a pattern formed by the combined influence of all the activity supportive attributes is associated with PA across countries. The stronger association for meeting the guideline by walking may have resulted from the greater influence and specificity of walkability design features (e.g. residential density, retail destinations, transit stops, sidewalks).

The class labeled high walkable and unsafe with few recreation facilities (Class 2) revealed the strongest association with meeting the guideline for total activity (OR = 1.62) and walking (OR = 2.26) relative to the most activity unsupportive (i.e., Class 5). However, large confidence intervals for both Classes 1 and 2 for total physical activity or walking suggest they may not differ from each other. Approximately, 16% of the sample across 11 countries was classified into this pattern with Colombia, Lithuania and Hong Kong overrepresented. In Class 2, multiple family homes and sidewalks were almost certain to exist as were strong probabilities for shops and transit stops within walking distance. Facilities supporting cycling and recreational physical activity were less likely to be reported within walking distance, and the sense of being unsafe at night was the greatest of all the classes. This combination of attributes suggests a typical urban walkable environment, which may explain the lower perceived safety from crime when population density is higher. However, high-density urban environments have been consistently associated with greater utilitarian or active transportation and less automobile dependence [9-12]. High residential densities provide the critical mass of people needed to support shops, services, and public transit. For example, it is well known that access to transit facilities vary by ridership, which is affected by residential density [12]. Additionally, sidewalks make up an important attribute of “complete streets” and provide a designated area for pedestrian travel. However, it was unexpected that this class, with greatest concerns about crime safety, would be associated with the greatest likelihood of meeting the physical activity guideline.

The relation of neighborhood crime to physical activity has been equivocal [10]. Across patterns, the results from this study found that perceived safe conditions in one’s neighborhood was not sufficient for higher levels of physical activity to occur in the absence of supportive built environment attributes for physical activity (i.e. Class 5). Yet, the greatest odds of meeting the guideline by walking occurred in the neighborhood typology with the highest perceived concerns of unsafe conditions, but any negative effect on physical activity appears to be mitigated by activity supportive features indicating a highly walkable urban design (i.e. Class 2). The neighborhood type with perceived presence of all seven built environment attributes, but equal likelihood of safety and unsafe conditions (i.e. Class 1), also was related to meeting the physical activity guideline. These results regarding personal safety were surprising and further add to the equivocal findings [18,46]. It seems intuitive that safe neighborhoods would be necessary for physical activity to occur. However, studies of lower income and minority populations in the US, which tend to live in poorer, dense, and more unsafe areas, suggest that prevalence of meeting the physical activity guideline does not differ by subgroup when physical activity is measured objectively [47], suggesting that transportation and occupational activities account for greater activity in these populations because active transportation occurred of necessity. Perhaps, even in the context of unsafe conditions, people adapt by learning behavioral responses (e.g. walking with groups, avoid eye contact), contextual cues (e.g. walking only during the day) or do not have a choice (e.g. need to go to work) that moderate the relations between unsafe conditions and activity-supportive environments and physical activity. Additionally, personal safety and neighborhoods type might be more salient for youth or older adult populations who are limited in their response to unsafe conditions. Research designed specifically to disentangle these complex effects is needed.

Class 3 (safe with active transport facilities) and Class 4 (transit and shops dense with few activity amenities) were not associated with meeting guideline, except that Class 4 was significantly and negatively associated with meeting the guideline for total activity. Class 3 appears to have attributes supportive of recreational physical activity, such as access to bicycle and low-cost recreational facilities, along with sidewalks, but because the IPS did not measure domain-specific physical activity, it unknown whether recreational activities would be associated with this class. It is unclear why Class 4 (transit and shops dense with few amenities) was negatively related to meeting the guideline for total physical activity. Perhaps a lack of recreation areas combined with safety concerns and lower residential densities, even if shops and transit are available, make this an aversive environment for recreational activities, which would tend to lower estimates of total physical activity.

Strengths of the current study included an international coordinated study with the use of samples of adults from representative urban areas in 11 countries; a validated and culturally adapted physical activity measure designed for international surveillance; a brief environment survey also designed for surveillance to assess key neighborhood attributes known to be associated with physical activity; and state-of-the science latent class analysis. Limitations included those inherent in a cross-sectional design such as the inability to disentangle cause-effect relationships and same source bias that can result from use of self-reports to assess both environment and physical activity. The IPAQ short form is global measure that may underestimate labor and domestic physical activities in developing countries. These reporting biases could be mitigated in the future by an international study collecting objective measures of built environment attributes and physical activity, such as the International Physical Activity and Neighborhood Environment (IPEN) Study [22], which will provide objective measures of both environment and activity. Additionally, seven single-item questions were used in the present study to assess several built environment constructs. These measures have shown fair-to-substantial agreement in a previous study [32] but may not capture the full range (e.g. intersection density) or depth of these environmental constructs. For example, measures of safety do not assess the type or severity of the dangers (e.g. property vs. violent personal crimes). While comprehensive self-report surveys of neighborhood design attributes are practical and affordable alternatives [48], numerous multiple-item scales were impractical for this international surveillance study. The PANES has shown acceptable reliability and validity compared to comprehensive multi-scale surveys of the built environment and physical activity [30,32]. Because educational attainment was unavailable for all countries, it is also possible that the generalized linear regression model is underspecified. Finally, cultural differences with respect to physical activity domains were not considered in this study.

Neighborhood design attributes supportive of physical activity vary both within and between countries. Previous analyses of the IPS environment data [30] and the current study suggested that individuals living in neighborhoods likely to have at least four attributes specific to walkability (i.e., access to shops and services, high residential densities, sidewalks, and transit stops) or that are “overall activity supportive” have a greater likelihood of meeting the PA guideline by walking or total activities. The WHO recommendation [1,5] for population-based strategies to increase physically active through improved urban design, public transportation, and recreation attributes was supported in this study of diverse countries.

## Competing interests

The authors declare that they have no competing interests.

## Authors’ contributions

MA conceptualized the paper and was the primary data analyst and writer of the paper. JFS, DD and HRB contributed to data management, analyses, drafting of the paper. AB and JFS conceived of the IPS study and participated in its design and coordination. BEA, PB, FCB, HC, CLC, IDB, LFG, MH, SI, LKH, JL, DJM, SM, VM, GM, NM, MS, HT, VV participated in the main study design, coordination, recruitment, study implementation in each country. All authors read, edited, and approved the final manuscript.

## References

[B1] World Health Organization Global status report on noncommunicable diseases 2010 Book Global status report on noncommunicable diseases 2010 (Editor ed.^eds.) 2011 WHO 1 161

[B2] Danaei G Ding EL Mozaffarian D Taylor B Rehm J Murray CJ Ezzati M The preventable causes of death in the United States: comparative risk assessment of dietary, lifestyle, and metabolic risk factors PLoS Med 2009 6 e1000058 10.1371/journal.pmed.1000058 19399161 PMC2667673

[B3] World Health Organization Global health risks: mortality and burden of disease attributable to selected major risks 2009 Geneva, Switzerland: World Health Organization

[B4] Guthold R Ono T Strong KL Chatterji S Morabia A Worldwide variability in physical inactivity a 51-country survey Am J Prev Med 2008 34 486 494 10.1016/j.amepre.2008.02.013 18471584

[B5] World Health Organization Interventions on diet and physical activity: what works: summary report 2009 Geneva: WHO 24432437

[B6] Sallis J Environmental and policy research on physical activity is going global Res Exerc Epidemiol 2011 13 111 117

[B7] Heath GW Brownson RC Kruger J Miles R Powell KE Ramsey LT Task Force on Community Preventive Services. The effectiveness of urban design and land use and transport policies and practices to increase physical activity: a systematic review J Phys Act Heal 2006 3 S55 S76 10.1123/jpah.3.s1.s55 28834525

[B8] Frank LD Sallis JF Saelens BE Leary L Cain K Conway TL Hess PM The development of a walkability index: application to the Neighborhood Quality of Life Study Br J Sports Med 2010 44 924 933 10.1136/bjsm.2009.058701 19406732

[B9] Saelens BE Sallis JF Frank LD Environmental correlates of walking and cycling: findings from the transportation, urban design, and planning literatures Ann Behav Med 2003 25 80 91 10.1207/S15324796ABM2502_03 12704009

[B10] Saelens BE Handy SL Built environment correlates of walking: a review Med Sci Sports Exerc 2008 40 S550 S566 10.1249/MSS.0b013e31817c67a4 18562973 PMC2921187

[B11] Durand CP Andalib M Dunton GF Wolch J Pentz MA A systematic review of built environment factors related to physical activity and obesity risk: implications for smart growth urban planning Obes Rev: an official journal of the International Association for the Study of Obesity 2011 12 e173 e182 10.1111/j.1467-789X.2010.00826.x PMC3079793 21348918

[B12] Ewing R Cervero R Travel and the Built Environment J Am Plan Assoc 2010 76 265 294 10.1080/01944361003766766

[B13] Kaczynski AT Henderson KA Parks and recreation settings and active living: a review of associations with physical activity function and intensity J Phys Act Health 2008 5 619 632 18648125 10.1123/jpah.5.4.619

[B14] Sallis JF Owen N Fisher E Glanz K, Rimer BK, Viswanath K Health Behavior and Health Education: Theory, Research, and Practice Health Behavior and Health Education: Theory, Research, and Practice. Volume 4th 2009 San Francisco: Jossey-Bass 465 482

[B15] Kaczynski AT Henderson KA Environmental correlates of physical activity: a review of evidence about parks and recreation Leisure Sci 2007 29 315 354 10.1080/01490400701394865

[B16] MacDonald JM Stokes RJ Cohen DA Kofner A Ridgeway GK The effect of light rail transit on body mass index and physical activity Am J Prev Med 2010 39 105 112 10.1016/j.amepre.2010.03.016 20621257 PMC2919301

[B17] Besser LM Dannenberg AL Walking to public transit: steps to help meet physical activity recommendations Am J Prev Med 2005 29 273 280 10.1016/j.amepre.2005.06.010 16242589

[B18] Foster S Giles-Corti B The built environment, neighborhood crime and constrained physical activity: an exploration of inconsistent findings Prev Med 2008 47 241 251 10.1016/j.ypmed.2008.03.017 18499242

[B19] Giles-Corti B Donovan RJ The relative influence of individual, social and physical environment determinants of physical activity Soc Sci Med 2002 54 1793 1812 10.1016/S0277-9536(01)00150-2 12113436

[B20] Wendel-Vos W Droomers M Kremers S Brug J van Lenthe F Potential environmental determinants of physical activity in adults: a systematic review Obes Rev: an official journal of the International Association for the Study of Obesity 2007 8 425 440 10.1111/j.1467-789X.2007.00370.x 17716300

[B21] Ding D Gebel K Built environment, physical activity, and obesity: what have we learned from reviewing the literature? Health Place 2012 18 100 105 10.1016/j.healthplace.2011.08.021 21983062

[B22] Kerr J Sallis JF Owen N De Bourdeaudhuij I Cerin E Reis R Sarmiento OL Frömel K Mitáš J Troelsen J Advancing Science and Policy through a Coordinated International Study of Physical Activity and Built Environments: IPEN Methods J Phys Act Healthin press 10.1123/jpah.10.4.581 22975776

[B23] Cerin E Saelens BE Sallis JF Frank LD Neighborhood environment walkability Scale: validity and development of a short form Med Sci Sports Exerc 2006 38 1682 1691 10.1249/01.mss.0000227639.83607.4d 16960531

[B24] Norman GJ Adams MA Kerr J Ryan S Frank LD Roesch SC A latent profile analysis of neighborhood recreation environments in relation to adolescent physical activity, sedentary time, and obesity J Public Health Manag Pract 2010 16 411 419 20689390 10.1097/PHH.0b013e3181c60e92 PMC3222690

[B25] Adams MA Sallis JF Kerr J Conway TL Saelens BE Frank LD Norman GJ Cain KL Neighborhood environment profiles related to physical activity and weight status: a latent profile analysis Prev Med 2011 52 326 331 10.1016/j.ypmed.2011.02.020 21382400 PMC3087437

[B26] Adams M Sallis J Conway T Frank L Saelens B Kerr J Cain K King A Neighborhood Environment Profiles Related to Physical Activity and Weight Status among Seniors: A Latent Profile Analysis Am J Heal Behav 2012 36 757 769 10.5993/AJHB.36.6.4

[B27] Wall MM Larson NI Forsyth A Van Riper DC Graham DJ Story MT Neumark-Sztainer D Patterns of obesogenic neighborhood features and adolescent weight: a comparison of statistical approaches Am J Prev Med 2012 42 e65 e75 10.1016/j.amepre.2012.02.009 22516505 PMC3380614

[B28] McDonald K Hearst M Farbakhsh K Patnode C Forsyth A Sirard J Lytle L Adolescent physical activity and the built environment: a latent class analysis approach Health Place 2012 18 191 198 10.1016/j.healthplace.2011.09.004 21975286 PMC3266467

[B29] Bauman A Bull F Chey T Craig CL Ainsworth BE Sallis JF Bowles HR Hagstromer M Sjostrom M Pratt M The International prevalence study on physical activity: results from 20 countries Int J Behav Nutr Phys Act 2009 6 21 10.1186/1479-5868-6-21 19335883 PMC2674408

[B30] Sallis JF Bowles HR Bauman A Ainsworth BE Bull FC Craig CL Sjostrom M De BI Lefevre J Matsudo V Neighborhood environments and physical activity among adults in 11 countries Am J Prev Med 2009 36 484 490 10.1016/j.amepre.2009.01.031 19460656

[B31] Forsyth A Oakes JM Schmitz KH Hearst M Does residential density increase walking and other physical activity? Urban Studies 2007 44 679 697 10.1080/00420980601184729

[B32] Sallis JF Kerr J Carlson JA Norman GJ Saelens BE Durant N Ainsworth BE Evaluating a brief self-report measure of neighborhood environments for physical activity research and surveillance: Physical Activity Neighborhood Environment Scale (PANES) J Phys Act Health 2010 7 533 540 20683096 10.1123/jpah.7.4.533

[B33] Alexander A Bergman P Hagströmer M Sjöström M IPAQ environmental module; reliability testing J Publ Health 2006 14 76 80 10.1007/s10389-005-0016-2

[B34] Oyeyemi AL Adegoke BO Oyeyemi AY Fatudimu BM Test-retest reliability of IPAQ environmental- module in an African population Int J Behav Nutr Phys Act 2008 5 38 10.1186/1479-5868-5-38 18680599 PMC2531132

[B35] Craig CL Marshall AL Sjostrom M Bauman AE Booth ML Ainsworth BE Pratt M Ekelund U Yngve A Sallis JF Oja P International physical activity questionnaire: 12-country reliability and validity Med Sci Sports Exerc 2003 35 1381 1395 10.1249/01.MSS.0000078924.61453.FB 12900694

[B36] U.S. Department of Health and Human Services 2008 Physical Activity Guidelines for Americans 2008 2008 Physical Activity Guidelines for Americans Washington DC: U.S. Department of Health and Human Services

[B37] Collins LM Lanza ST Latent Class and Latent Transition Analysis with Application in the Social, Behavioral, and Health Sciences 2010 Hoboken, New Jersey: John Wiley & Sons

[B38] Akaike H Factor-Analysis and Aic Psychometrika 1987 52 317 332 10.1007/BF02294359

[B39] Schwarz G Estimating the dimension of a model Ann Stat 1978 6 461 464 10.1214/aos/1176344136

[B40] Frank LD Engelke PO Schmid TL Health and community design: the impact of the built environment on physical activity 2003 Washington, DC: Island Press

[B41] Sallis JF Floyd MF Rodriguez DA Saelens BE Role of built environments in physical activity, obesity, and cardiovascular disease Circulation 2012 125 729 737 10.1161/CIRCULATIONAHA.110.969022 22311885 PMC3315587

[B42] Sallis JF Saelens BE Frank LD Conway TL Slymen DJ Cain KL Chapman JE Kerr J Neighborhood built environment and income: examining multiple health outcomes Soc Sci Med 2009 68 1285 1293 10.1016/j.socscimed.2009.01.017 19232809 PMC3500640

[B43] Frank LD Schmid TL Sallis JF Chapman J Saelens BE Linking objectively measured physical activity with objectively measured urban form Findings from SMARTRAQ Am J Prev Med 2005 28 117 125 10.1016/j.amepre.2004.11.001 15694519

[B44] Gebel K Bauman A Owen N Correlates of non-concordance between perceived and objective measures of walkability Ann Behav Med 2009 37 228 238 10.1007/s12160-009-9098-3 19396503

[B45] Adams MA Ryan S Kerr J Sallis JF Patrick K Frank L Norman GJ Validation of the Neighborhood Environment Walkability Scale (NEWS) Items Using Geographic Information Systems J Phys Act Heal 2009 6 S113 S123 10.1123/jpah.6.s1.s113 19998857

[B46] Loukaitou-Sideris A Eck JE Crime prevention and active living Am J Health Promot 2007 21 380 389 iii 10.4278/0890-1171-21.4s.380 17465184

[B47] Troiano RP Berrigan D Dodd KW Masse LC Tilert T McDowell M Physical activity in the United States measured by accelerometer Med Sci Sports Exerc 2008 40 181 188 18091006 10.1249/mss.0b013e31815a51b3

[B48] Saelens BE Sallis JF Black JB Chen D Neighborhood-based differences in physical activity: an environment scale evaluation Am J Public Health 2003 93 1552 1558 10.2105/AJPH.93.9.1552 12948979 PMC1448009

